# The Social Validity of Behavioral Interventions: Seeking Input from Autistic Adults

**DOI:** 10.1007/s10803-024-06297-3

**Published:** 2024-03-12

**Authors:** Kaitlynn M.P. Baiden, Zachary J. Williams, Rachel K. Schuck, Patrick Dwyer, Mian Wang

**Affiliations:** 1 Gevirtz Graduate School of Education, University of California, Santa Barbara, USA; 2 Medical Scientist Training Program, Vanderbilt University School of Medicine, Nashville, USA; 3 Department of Hearing and Speech Sciences, Vanderbilt University Medical Center, Nashville, USA; 4 Vanderbilt Brain Institute, Vanderbilt University, Nashville, USA; 5 Frist Center for Autism and Innovation, Vanderbilt University, Nashville, USA; 6 Vanderbilt Kennedy Center, Vanderbilt University Medical Center, Nashville, USA; 7 Department of Psychology, University of California, Davis, Davis, USA; 8 Center for Mind and Brain, University of California, Davis, Davis, USA; 9 Olga Tennison Autism Research Centre (OTARC), School of Psychology and Public Health, La Trobe University, Bundoora, VIC Australia

**Keywords:** Social validity, Behavioral interventions, Autistic adults, Naturalistic developmental behavioral intervention

## Abstract

Many in the autistic community have expressed concerns regarding the use of behavioral interventions with autistic children, suggesting that these interventions may not be socially valid. Though behavioral interventions have evolved to be more naturalistic and child-centered, little structured research has been done to explicitly seek autistic perspectives on the acceptability of specific components of behavioral interventions. Autistic adults (*N* = 235) were recruited online to take the Autism Intervention Attitudes Scale (AIAS), a questionnaire designed to gather feedback on common intervention goals and practices. Results indicate that participants find goals and practices that highlight quality of life, safety, and autistic interactions acceptable, while those that focus on normalization based on neurotypical standards are not. An exploratory graph analysis revealed three communities of goals (“uncontroversial goals”, “controversial goals”, and “social goals”). Comparison between naturalistic and structured intervention components additionally showed that autistic participants favored naturalistic strategies. These findings are in line with known criticisms of behavioral intervention from autistic adults, but also provide more information on the specific ways in which behavioral interventions can be reformed. This information can guide professionals in the development of appropriate goals and decisions around intervention planning.

Behavioral intervention programs for autistic children have been widely used for the past three decades (Wergeland et al., 2022). These models are based on principles of behaviorism where reinforcement procedures are used to teach new skills and reduce unwanted behaviors. These intervention models originally gained popularity in the autism field after the publication by Ivaar Lovaas in 1987. Lovaas’ study proclaimed that 40 h a week of intensive behavioral intervention was effective in reducing autism “symptoms,” so much to say that over half of the study’s participants were “indistinguishable from their normal [peers]” (p. 8). Though this study has been heavily criticized for its misleading results (Ospina et al., 2008; Sandbank et al., 2020), it was successful in spurring the use of early intensive behavioral intervention for young autistic children, further propelling the field of Applied Behavior Analysis (ABA), which has since become one of the most utilized services for autistic children (Monz et al., 2019). This paper discusses the concept of social validity as it pertains to behavioral interventions, the lack of autistic voices in behavior analytic research, and common concerns that autistic people have voiced when it comes to the use of ABA interventions. Based on this rationale, this study aims to gain the input of autistic adults on specific aspects of behavioral interventions that are commonly used and that are related to the critiques of ABA.

One key aspect of all behavioral interventions is the intervention’s social validity— stakeholders’ opinions regarding the social significance of intervention goals, the social appropriateness of intervention procedures, and the social importance of intervention effects (Wolf, 1978). Over the past four decades, researchers have emphasized the importance of social validity from both ethical standpoints (i.e., participants have a right to have a say in the interventions they receive; Hanley, 2010; Wolf, 1978) and program evaluation perspectives (i.e., programs that are acceptable are more likely to be adopted and sustained; Schwartz & Baer, 1991; Wolf, 1978). Furthermore, social validity has been established as a criterion for an intervention being considered “evidence-based” (Horner et al., 2005; Reichow, 2011). However, multiple reviews have shown that the use of social validity assessments in behavioral intervention research is infrequent (e.g., Callahan et al., 2017; D’Agostino et al., 2019; Ledford et al., 2016), and even when it occurs, intervention goals are rarely evaluated (D’Agostino et al., 2019; Ferguson et al., 2019). Furthermore, disabled participants themselves are rarely involved in the social validation process (D’Agostino et al., 2019; Hurley et al., 2012; Monahan et al., 2023).

Given this lack of social validation, particularly from the autistic perspective, it is perhaps not surprising that behavioral intervention has been the subject of harsh criticism from many autistic adults (for reviews from different perspectives, see Chapman & Bovell, 2022; Graber & Graber, 2023; Leaf et al., 2022; Schuck et al., 2021). Individuals report trauma as a result of such interventions, highlighting three main concerns: the use of aversives to change behavior both in terms of historical use and in some cases continued use of extreme punishments such as hitting or electric shocks (e.g. Simmons & Lovaas, 1969) and more currently accepted practices such as encouraging “quiet hands” (Bascom, 2011), its focus on compliance over quality of life (e.g., Sandoval-Norton & Shkedy, 2019), and its emphasis on “normalization” as opposed to acceptance (Gardner, 2017; McGill & Robinson, 2021; Stop ABA, Support Autistics, 2019; also see Gibson & Douglas, 2018, for a discussion on how ABA has been used in homosexual conversion therapy). Many feel that behavioral intervention promotes masking or camouflaging—the suppression of autistic traits to fit into neurotypical society (Bargiela et al., 2016; Hull et al., 2017)—by focusing on changing the autistic person’s behavior, communication style, and mannerisms in such a way that they look less autistic (e.g., by promoting varied conversation instead of focusing on preferred topics or reducing hand flapping). The link between behavioral intervention and masking is particularly concerning given that masking has been correlationally linked to negative mental health outcomes (Cage & Troxel-Whitman, 2019). These concerns and critiques are directly related to social validity. Concerns regarding the use of aversive punishment and compliance-based techniques tap directly into the acceptability of intervention procedures, whereas concerns about normalization map onto the acceptability of intervention goals.

In the decades since Lovaas’ (1987) influential study, the field of behavioral intervention has attempted to rectify some of its most glaring issues. Various ABA intervention models have evolved to become more naturalistic and client-centered, although many community-based interventionists appear to be poorly informed about many of these developments (Hampton & Sandbank, 2022). The initial Lovaas study evaluated the effectiveness of an intervention model known as Discrete Trial Training (DTT; Smith, 2001). DTT focuses on teaching discrete skills one at a time using repetitive trials and external reinforcement. However, due to the time-consuming nature of this intervention, lack of skill generalization, and low motivation observed in children (Koegel et al., 1998), interventions were adapted to be more naturalistic and motivation-based; these would later be termed Naturalistic Developmental Behavioral Interventions (NDBIs; Schreibman et al., 2015). These models focus on following the child’s lead and using more naturalistic methods of reinforcement, thus emphasizing motivation and skill generalization. While NDBIs are theoretically different from DTT, DTT has also gradually become more naturalistic as well (Ferguson & Milne, 2023; Leaf et al., 2017). As such, from the perspective of many professionals, researchers, and clinicians, ABA-based interventions have drastically improved and become more socially valid. This perspective is not universally shared; in response to autistic and neurodiversity movement critiques of ABA, increasing discussion centers on how to further improve interventions to be more neurodiversity-affirming (Donaldson et al., 2017; Leadbitter et al., 2021; Schuck et al., 2021). However, as discussed above, research on behavioral intervention has rarely included autistic perspectives when assessing social validity; thus, much work remains to ensure more neurodiversity-affirming interventions are used. Recent evidence suggests that even NDBIs are not entirely socially valid. For example, after watching short clips of adults using a common NDBI, Pivotal Response Treatment (Koegel et al., 2016), to encourage spoken language in autistic children, many autistic adults stated that they felt that prioritizing spoken language above other communication methods was not necessary and could even be detrimental (Schuck et al., 2022). Though participants highlighted positive aspects of the intervention, such as adults reinforcing children’s attempts to communicate, they also had other concerns regarding compliance and ignoring children’s feelings (Schuck et al., 2022).

Though autistic individuals have been clear about their concerns regarding normalization and compliance, and autistic advocates have even offered guidance regarding many specific goals and practices (e.g., Autistic SelfAdvocacy Network, 2021), autistic perspectives regarding many specific aspects of behavioral intervention have yet to be included in empirical research studies (Leaf et al., 2022). That is, what intervention goals and practices are problematic and contribute to this culture of forced “normalization” and teaching compliance? In fact, a recent study evaluating the appropriateness of support goals for autistic children from the perspective of parents, professionals, and autistic adults in New Zealand and Australia, is the first study we are aware of to include the autistic perspective on the topic of intervention goals (Waddington et al., 2023). The lack of inclusion of autistic perspectives on program goals and practices in research is troublesome as we attempt to increase social validity and reconcile differences between professionals aiming to support autistic individuals and autistic individuals themselves. It is critical that researchers begin to ask for explicit feedback from autistic individuals to determine if behavioral interventions can be rectified and, if so, how they can be improved to best support the autistic population in a way that is significant to them.

## Current Study

This study aimed to gain autistic adults’ feedback on common intervention goals and practices via an online survey. Our research questions were as follows:

1. Which common behavioral intervention goals are seen as socially valid by autistic adults?
  - Is there a logical way of grouping goals such that providers can more easily assess the goal’s social validity?
2. Which common behavioral intervention practices and procedures are seen as socially valid by autistic adults?
  - Do autistic adults prefer certain intervention practices over others?

## Method

### Procedure

This study was approved by the University of California, Santa Barbara Institutional Review Board. Study advertisements were sent to autism organizations and posted on social media pages and groups meant for autistic individuals. Groups espousing a variety of views on ABA were contacted including groups for autistic behavior analysts and groups that explicitly stated an anti-ABA stance. All participants gave informed consent before beginning the survey. The study was conducted via Qualtrics. Individuals needed to be autistic (formally diagnosed or self-identified) and at least 18 years old to participate. Participants first completed the Ritvo Autism Asperger Diagnostic Scale—Screen (RAADS-14; Eriksson et al., 2013). The RAADS-14, a validated 14-item autism diagnostic screener, was not used as a means of exclusion in this study, but it was used to describe the sample of participants. Next, participants were directed to the study survey.

#### Autism Intervention Attitudes Scale

Participants were presented with the Autism Intervention Attitudes Scale (AIAS), a novel 41-item survey created by the research team that included 19 statements about intervention goals and 22 statements about intervention practices and procedures, four of which explicitly describe NDBI strategies. The items about practices and procedures included statements about specific intervention components (e.g., naturalistic reinforcement, external reinforcement) and details about broader aspects of intervention (e.g., who decides on intervention goals, where they are conducted, intervention priorities). These items were written by the authors of this manuscript for the purpose of this study and were selected based on researchers’ experience with providing behavioral intervention, review of common intervention practices/procedures and goals in the intervention literature, and common critiques of intervention practices/procedures and goals in autistic self-advocate forums (e.g., social media groups, blogs, etc.). The first four authors brainstormed initial items based on lived experience, expertise, and literature review. To maximize the content validity of the measure, the research team added items independently and discussed them during team meetings until a consensus was reached regarding which items were most relevant to the practical implementation of interventions for autistic children. It is important to note the positionality of the authors who contributed to the creation of these items. Two of these authors identify as autistic (ZJW and PD), one has experience providing and supervising behavior analytic services in a community agency and in a research setting (KMPB), and the final author has experience implementing NDBIs in university research and clinical settings (RKS), providing a range of experiences and knowledge on the topic. All authors approved the items in the survey. A full list of the survey items is included in the Supplementary Material. Participants were asked to rate the degree to which they agreed with each statement using a 6-point Likert scale (1 = *strongly disagree*; 6 = *strongly agree*). They were also provided an optional open-ended text box where they could expand upon their answer (due to the amount of qualitative data, these data are not reported herein).

### Participants

A total of 235 autistic individuals (120 female, 43 male, 51 nonbinary/genderqueer, 21 other/unknown; mean age = 34.36 [*SD* = 11.13]) responded to at least some of the AIAS. 150 participants (63.8%) lived in the United States, 60 lived elsewhere, and 25 did not specify. Most participants identified as White (*n* = 193; 82.1%). Seventy (29.8%) indicated that they had received behavioral intervention during childhood, 111 reported that they did not, and 45 were unsure. Most (*n* = 178; 75.7%) indicated a clinical autism diagnosis (average age of diagnosis = 24.87 years [*SD* = 13.10]), while 57 identified as autistic but did not have a diagnosis. RAADS-14 scores ranged from 6 to 42 (*M* = 32.29, *SD* = 6.92); only four participants scored below the autism cutoff of 14, all of whom reported a formal diagnosis (the RAADS was incomplete for two participants).

### Data Analysis

#### Intervention Goals

To assess the social validity (i.e., acceptability) of intervention goals, two types of descriptive statistics are reported for each goal statement: (1) the percentage of participants who endorsed the goal (i.e., they slightly agreed/agreed/strongly agreed that it was a good intervention goal); and (2) the mean rating of each goal (closer to 1 indicating strong disagreement that it is a good goal; closer to 6 indicating strong agreement that it is a good goal).

An exploratory graph analysis (EGA; Golino & Epskamp, 2017; Golino et al., 2020) was used to examine the structure of intervention goals and cluster the goals into a smaller number of dimensions for further analysis. The EGA algorithm was implemented in the *EGAnet* R package (Golino & Christensen, 2021) using polychoric correlations, EBICglasso network estimation, the Walktrap community-finding algorithm, and the leading eigenvalue method for unidimensionality assessment (Christensen et al., 2021). After conducting the EGA, each goal was assigned to a specific “community” (i.e., a cluster of variables that form a latent factor) allowing us to analyze the number of goals endorsed within each community as a composite score reflecting the level of agreement or disagreement with that goal cluster.

Hierarchical Bayesian logistic regression models were used to predict the number of goals endorsed within each goal cluster generated by the EGA. Each goal outcome within the cluster was dichotomized into “endorsed” (any level of agreement) or “not endorsed” (any level of disagreement) and analyzed within a hierarchical framework that treated “individual” and “goal” as crossed random effects. Fixed effects in all models included participant age, gender (male [baseline] vs. female vs. non-binary/other), diagnostic status (formal autism diagnosis vs. self-diagnosed), RAADS-14 score, and prior behavioral intervention experience (yes vs. no/not sure), and all random slopes were modeled by item, allowing each predictor variable to differentially influence the endorsement of each goal within the cluster. Weakly-informative priors were placed on all parameters, including a default *t*_3_(0, 2.5) prior on the intercept term, a Normal(0, 1) prior on all standardized regression slopes, a half-*t*_3_(0, 2.5) prior on all random effect standard deviations, and a Lewandowski-Kurowicka-Joe (2009) prior (𝜂 = 2) on the random-effect correlation matrix. All Bayesian regression models were fit in Stan (Carpenter et al., 2017) using Hamiltonion Monte Carlo, as implemented in the *brms* R package (Bürkner, 2017, 2018).

Fixed effects drawn from the Bayesian models were converted to odds ratios (ORs), analogous to those in standard frequentist logistic models. For continuous predictors (e.g., age, RAADS-14 score), the OR was the exponentiated standardized beta coefficient, the increased odds of goal endorsement with a 1 standard deviation increase in the predictor. Within each Bayesian regression model, OR values were tested against the interval null hypothesis that the true OR lies within the interval [0.833, 1.2], termed the Region of Practical Equivalence (ROPE; Kruschke & Liddell, 2018). This region contains all values that were determined a priori to be too small to be meaningful, even if the true OR is not strictly equal to 1. The ROPE Bayes factor (*BF*_ROPE_; Linde et al., 2023; Makowski et al., 2019) was used to quantify evidence for or against the null hypothesis, with values greater than 3 suggesting substantial evidence in favor of the alternative hypothesis, and values less than ^1/3^ providing substantial evidence in favor of the null hypothesis (Wagenmakers et al., 2011). Additional Bayesian indices of effect significance included the probability of direction (*P*_d_, i.e., the posterior probability of the population odds ratio being greater than 1 [in the case of OR > 1] or less than 1 [in the case of OR < 1]; Makowski et al., 2019), the ROPE probability (*P*_ROPE_; Liao et al., 2021; i.e., the posterior probability that the population OR value falls within the null region [0.833, 1.2]), and the probability of practical significance (*P*_PS_, i.e., the posterior probability of the population odds ratio exceeding the ROPE in the direction of the point estimate).

#### Intervention Practices and Procedures

Acceptability of intervention practices/procedures was assessed by reviewing descriptive statistics for each practice statement. Similar to intervention goals, participants were considered endorsing an intervention practice if they somewhat agreed, agreed, or strongly agreed with the statement. The percentage of participants who endorsed each practice, as well as the mean degree to which participants agreed with the practice described, are reported.

To assess whether participants preferred certain intervention procedures over others, potentially-contrasting practices were grouped together a priori (e.g., natural rewards versus external rewards; see results for all groupings), and differences in ratings were assessed using hierarchical Bayesian ordered-probit regression models (Bürkner & Vuorre, 2019) with a fixed effect of item, random intercept by individual, and residual variance term allowed to vary according to each item. Priors for these models included a *t*_3_(0, 2.5) prior on all intercept terms, a Normal(0, 1) prior on fixed effects, a half-*t*_3_(0, 2.5) prior on all random effect standard deviations, and a uniform prior on the inverse of the residual standard deviation parameter for each item. The latent mean and variance of the baseline item were respectively set to 0 and 1 for model identification. Within-person comparisons between items were quantified using Cohen’s *d*, scaled by the pooled (latent scale) variance of all items assessed in the model. A posterior probability of 0.95 or greater that the effect size was larger than *d* = ± 0.5 (i.e., more than half a standard deviation difference between responses to each item) was used as the criterion for a practically meaningful difference in ratings between two items.

#### Community Involvement Statement

Two authors of this article (ZJW and PD) identify as autistic. These authors have been involved in all aspects of the study, including conceptualization of the study, development of the AIAS items, data collection and analysis, and writing and editing the manuscript.

## Results

### Intervention Goals

#### Descriptive Statistics

**Ten** of 19 goals were endorsed by most participants. Almost all participants (96%) felt that reducing dangerous situations was an important goal, and this goal received the highest mean rating (5.4/6; see Table 1 for descriptive statistics). Over 90% also felt that improving communication skills, improving quality of life, and reducing self-injurious behaviors were also good intervention goals. Fewer than 20% of participants felt that reducing vocal or physical self-stimulatory behaviors and fixations were good goals, with the latter two receiving the lowest mean rating (1.7/6). Furthermore, reducing stimming and fixations, along with increasing eye contact, received a “strongly disagree” rating from over half the sample.

**Table 1:** Community Structure of intervention goals derived from exploratory graph analysis

Communities	% Endorsed	Mean (SD)
**Uncontroversial**		
Reducing Danger	96.2	5.4 (1.0)
Reducing Self-Injurious Behavior	90.9	5.1 (1.2)
Improving Quality of Life	91.3	5.3 (1.2)
Increasing Independence	88.3	4.7 (1.3)
Toileting	87.9	4.8 (1.3)
**Controversial**		
Reducing Inattention/Hyperactivity	49.1	3.3 (1.5)
Improving Sensory Tolerance	36.7	2.7 (1.7)
Reducing Picky Eating	32.2	2.7 (1.5)
Reducing Noncompliance	26.8	2.4 (1.6)
Increasing Eye Contact	20.1	2.0 (1.5)
Reducing Vocal Stimming*	19.0	2.0 (1.4)
Reducing Motor Stimming*	11.9	1.7 (1.3)
Reducing Fixations	9.0	1.7 (1.2)
**Social**		
Improving Communication Skills	91.5	4.9 (1.2)
Improving Interpersonal Skills	80.3	4.5 (1.4)
Learning Rules of Interaction	68.8	4.0 (1.5)
Improving Conversation Ability	63.6	3.9 (1.5)
Increasing School Readiness	49.1	3.3 (1.6)

*Note* *These two items were combined for the purposes of EGA

#### Exploratory Graph Analysis

Prior to conducting EGA analysis of the 19 intervention goals, we examined the magnitudes of pairwise polychoric correlations to determine whether any of the listed goals appeared redundant with one another (defined as two items sharing ≥ 70% of their variance, i.e., *r* ≥ 0.837). Based on this criterion, goals regarding the reduction of repetitive motor movements and repetitive vocalizations correlated highly (*r*_poly_ = 0.873) and were therefore summed together into a single 11-point “super-item” for the purposes of EGA. After combining these items together, no additional item pairs met our criteria for redundancy, and the remaining items were subjected to EGA. Based on an EGA of polychoric correlations, intervention goals clustered into three communities (see Table 1 for full community assignments), which we interpreted as “Uncontroversial Goals” (e.g., reducing danger, increasing independence), “Controversial Goals” (e.g., increasing eye contact, reducing motor/vocal stimming), and “Social Goals” (e.g., improving conversational ability, learning rules of social interaction). Intervention goal endorsement within each of these clusters was then examined in the context of cluster-specific hierarchical logistic regressions.

#### Comparison of Goal Endorsement by Demographic Groups

To explore whether demographic variables were related to goal endorsement, three hierarchical Bayesian logistic regressions were run (for Uncontroversial, Controversial, and Social goals, respectively). Each model regressed dichotomous goal endorsement onto age, gender, diagnostic status, RAADS-14 score, and prior behavioral intervention status. Overall, demographic variables had few meaningful associations on goal endorsement (see Table 2). The only relationship that exceeded the threshold for practical significance was that of gender, which significantly predicted endorsement of both Uncontroversial and Social goals. More specifically, individuals who identified as non-binary/other gender were significantly *less* likely to endorse both goal types (Uncontroversial: OR = 0.189, CrI_95%_ [0.060, 0.625], *P*_d_ = 0.995, *BF*_ROPE_ = 22.9; Social: OR = 0.247, CrI_95%_ [0.065, 1.057], *P*_d_ = 0.965, *BF*_ROPE_ = 4.94) than the reference group (men). Non-binary individuals demonstrated a trend toward endorsing fewer Controversial goals as well, although this did not reach the threshold for practical significance (OR = 0.389, CrI_95%_ [0.112, 1.370], *P*_d_ = 0.929, *BF*_ROPE_ = 2.17). ROPE Bayes factors for all remaining predictors provided inconclusive evidence for or against the interval null hypothesis, with the exception of RAADS-14 score, which was found to conclusively not predict Social goal endorsement to a meaningful extent (i.e., significant evidence for the null; OR = 0.840, CrI_95%_ [0.511, 1.386], *P*_d_ = 0.763, *BF*_ROPE_ = 0.219).

**Table 2:** Odds-ratios and bayesian indices for regression models predicting goal endorsement

	OR [95% CrI]	P_d_	P_ROPE_	P_PS_	BF_ROPE_
**Uncontroversial Goals**					
Age	0.717 [0.458, 1.130]	0.928	0.242	0.745	0.536
Female Gender	0.787 [0.302, 2.129]	0.682	0.257	0.544	0.504
**Non-binary Gender**	**0.189 [0.060, 0.625]**	**0.995**	**0.007**	**0.991**	**22.9**
Self-Diagnosed	1.000 [0.377, 2.438]	0.500	0.299	0.352	0.409
Received BI as Child	1.705 [0.664, 4.252]	0.868	0.161	0.772	0.875
RAADS-14 Score	0.705 [0.447, 1.084]	0.943	0.216	0.775	0.616
**Controversial Goals**					
Age	0.592 [0.334, 1.016]	0.968	0.093	0.897	1.630
Female Gender	0.850 [0.277, 2.656]	0.616	0.248	0.485	0.518
Non-binary Gender	0.389 [0.112, 1.370]	0.929	0.073	0.885	2.173
Self-Diagnosed	1.081 [0.366, 3.224]	0.559	0.262	0.424	0.472
Received BI as Child	0.835 [0.304, 2.149]	0.649	0.279	0.498	0.444
RAADS-14 Score	0.585 [0.318, 1.046]	0.967	0.099	0.890	1.636
**Social Goals**					
Age	0.661 [0.280, 1.581]	0.843	0.201	0.718	0.665
Female Gender	0.617 [0.192, 2.051]	0.789	0.167	0.694	0.865
**Non-binary Gender**	**0.247 [0.065, 1.057]**	**0.965**	**0.034**	**0.945**	**4.94**
Self-Diagnosed	0.698 [0.256, 1.884]	0.763	0.222	0.636	0.591
Received BI as Child	1.537 [0.601, 4.162]	0.819	0.198	0.702	0.678
*RAADS-14 Score*	*0.840 [0.511, 1.386]*	*0.763*	*0.436*	*0.488*	*0.219*

*Note* All continuous predictors are standardized coefficients (i.e., OR represents 1 SD increase in the variable). Predictors with substantial evidence for a practically significant effect are presented in bold, whereas predictors with substantial evidence against a practically significant effect are presented in italics. OR = Odds Ratio; CrI = Highest Density Credible Interval; *P*_d_ = Probability of Direction; *P*_ROPE_ = Probability of the OR falling within the ROPE; *P*_PS_ = Probability of practically significant effect; *BF*_ROPE_ = ROPE Bayes factor; BI = behavioral intervention; RAADS-14 = 14-item Ritvo Autism Asperger Diagnostic Scale—Screen

### Intervention Practices and Procedures

#### Descriptive Statistics

Almost all participants (> 99%) agreed that interventionists should try to see the world from the child’s perspective and that they should promote any communication technique that works (these were not endorsed by only 1 and 2 participants, respectively), with both items receiving mean ratings of 5.7/6 (see Table 3 for descriptive statistics). The least endorsed practice (promoting communication primarily via verbal speech) was only endorsed by 13.1% of participants and received the lowest mean rating (1.8/6).

**Table 3:** Endorsement and mean ratings of intervention practices and procedures

Practice	% Endorsed	Mean (SD)
Interventionists should try to see the world from the child’s perspective	99.6	5.7 (0.6)
Interventionists should promote communication using any technique that works (for example, sign language, pointing to symbols, speech generating devices)	99.1	5.7 (0.6)
Autistic adults should be consulted when intervention goals for a young child are developed	96.1	5.6 (0.9)
Autistic children who can communicate their needs should play a leading role in the development of intervention goals	95.2	5.6 (1.0)
When talking to parents, interventionists should emphasize autistic children’s strengths	94.2	5.3 (1.0)
Interventionists should use the autistic child’s interests to teach them new skills	93.0	5.3 (1.2)
Interventionists should focus on autistic children’s internal thoughts and feelings	92.4	5.2 (1.2)
Interventions should occur in natural settings, like a child’s home	84.3	4.6 (1.2)
Interventions should occur at school, as part of the child’s typical school day	76.1	4.1 (1.4)
Intervention services should be provided by non-profit organizations	74.9	4.3 (1.4)
Trained professionals should play a leading role in the development intervention goals	65.4	3.8 (1.6)
Interventions should be primarily delivered by trained professionals, like doctors and therapists	62.6	3.9 (1.4)
Parents should play a leading role in the development of intervention goals	61.9	3.8 (1.5)
Interventionists should teach children new skills in such a way that practicing the skill naturally leads to a reward (for example, letting the child play with a ball if they say “ball”)	61.5	3.8 (1.7)
Interventionists should try to engage with autistic children by imitating their behaviors	60.3	3.8 (1.4)
Interventions should occur in clinical settings, like a doctor’s office or speech therapy clinic	49.3	3.4 (1.4)
Interventionists should use rewards such as candy or stickers to teach children new skills (for example, giving the child a piece of candy if they can correctly label flashcards)	35.1	2.7 (1.7)
Interventions should be primarily delivered by teachers	34.4	2.9 (1.4)
When talking to parents, interventionists should emphasize autistic children’s challenges	32.6	3.0 (1.6)
Interventionists should focus on autistic children’s external behaviors	29.8	2.7 (1.5)
Intervention services should be provided by for-profit organizations	26.9	2.5 (1.5)
Interventionists should promote communication by focusing primarily on verbal speech	13.1	1.8 (1.3)

#### Intervention Practice and Procedures Preferences

Building on the raw descriptive agreement statistics (see Table 3), participant preferences for eleven potentially-contrasting intervention practices and procedures were evaluated (see Table 4 for full results). Participants preferred natural rewards over external rewards;^1^ promotion of any communication method over focusing on verbal speech; child-chosen goals over parent-chosen and professional-chosen goals; interventions at home over at a clinic; clinician-delivered interventions over teacher-delivered interventions; non-profit providers over for-profit providers; and focusing on internal thoughts and feelings over focusing on observable behaviors. While participants rated interventions at home higher than interventions at school and clinician-delivery higher than parent-delivery, these differences were not practically significant based on our *a priori* effect size criteria.

**Table 4:** Comparison of intervention practice and procedure preferences

Practice 1	Practice 2	d [95% CI]	P_(d>0.5)_
Natural Rewards	External Rewards	1.002 [0.764, 1.232]	> 0.999*
Promotion of Any Communication Method	Focus on Verbal Communication	2.939 [2.587, 3.282]	> 0.999*
Child-Chosen Goals	Parent-Chosen Goals	2.111 [1.827, 2.386]	> 0.999*
Child-Chosen Goals	Professional-Chosen Goals	2.134 [1.851, 2.416]	> 0.999*
Parent-Chosen Goals	Professional-Chosen Goals	0.025 [-0.110, 0.163]	< 0.001
Interventions at Home	Interventions at Clinic	1.082 [0.875, 1.301]	> 0.999*
Interventions at Home	Interventions at School	0.498 [0.289, 0.695]	0.511
Interventions at Clinic	Interventions at School	0.586 [0.394, 0.775]	0.813
Parent-Delivery	Clinician-Delivery	-0.256 [-0.039, -0.457]	0.012
Parent-Delivery	Teacher-Delivery	0.675 [0.495, 0.864]	0.969*
Clinician-Delivery	Teacher-Delivery	0.930 [0.718, 1.136]	> 0.999*
Non-Profit Providers	For-Profit Providers	1.307 [1.072, 1.537]	> 0.999*
Focus on Internal Feelings	Focus on Observable Behaviors	1.800 [1.564, 2.037]	> 0.999*

*Note* Cohen’s *d* values are calculated from within-person latent mean comparisons in a Bayesian hierarchical ordered-probit regression model. Positive *d* values favor Practice 1, whereas negative *d* values favor Practice 2

* Practically significant contrast (*P*_(*d*>0.5)_ > 0.95)

## Discussion

It is necessary for any intervention to assess social validity—stakeholders’ views on intervention goals, procedures, and outcomes—as it can ensure programs are both respectful to recipients and effective in the long-term. Though social validity is recognized as an integral part of behavioral intervention programs (Horner et al., 2005; Reichow, 2011), it has not been assessed in autism research studies nearly as often as it should be (Callahan et al., 2017; D’Agostino et al., 2019; Snodgrass et al., 2018). Even when it is assessed, ratings usually come from parents or interventionists, not autistic individuals themselves. Moreover, social validity questionnaires are generally provided to parents by the clinicians and research teams providing the service, which can result in social desirability bias. This disconnect could be part of the reason why many in the autistic community do not find ABA interventions acceptable (Cumming et al., 2020; Graber & Graber, 2023) despite the prominence of ABA as an intervention modality for autistic individuals (Monz et al., 2019). Our findings touch upon two of the three dimensions of social validity set forth by Wolf (1978): appropriateness of intervention goals and acceptability of intervention procedures. Our analysis helps identify which intervention goals, practices, and procedures are likely seen as important and acceptable to the autistic community more broadly, which are likely viewed more unfavorably, and which require a more nuanced investigation.

### Social Validity of Intervention Goals

*Uncontroversial* goals—those that were highly endorsed by participants—centered around safety and promotion of quality of life. It is perhaps unsurprising that these goals were highly endorsed, as many of these goals would likely be applicable to all children, regardless of diagnosis (e.g., toileting skills, reducing dangerous situations, increasing independence). Furthermore, this finding is in line with Waddington and colleagues’ (2023) preliminary research on the topic, which is especially encouraging given that their research was done in Australia while most of the participants in the current study were from the United States. While intervention goals should always be determined in consultation with clients and their families, it is likely that *uncontroversial* goals will be socially valid to many autistic clients across the spectrum. Clinicians should ensure that these types of goals are addressed through ongoing discussions with clients and families.

*Controversial* goals, on the other hand, were not endorsed by most participants and had very low mean ratings (thus, they arguably have limited social validity). These goals focused on reducing behaviors often associated with autism, such as stimming and atypical eye contact. These findings are in line with prior qualitative research that indicates many autistic people enjoy having the ability to stim if it is not hurting anyone (Kapp et al., 2019) and that forced eye contact can cause discomfort and is not considered necessary for communication (Dalmayne, 2017; Trevisan et al., 2017). Moreover, this corroborates the concern that many autistic individuals have regarding ABA intervention’s focus on normalization over quality of life (Gardner, 2017; McGill & Robinson, 2021; Stop ABA, Support Autistics, 2019). Clinicians and educators should be wary of such goals and only include them in an intervention plan if there is a reason to believe it will significantly improve quality of life and/or the client themselves is interested in doing so (indeed, these goals were all endorsed by a minority of participants). For example, a client may wish to improve their tolerance of certain sounds that are both uncomfortable and unavoidable, if those sounds are impeding their school or work performance. Similarly, if an autistic person finds their idiosyncratic interests are becoming so absorbing that they interfere with schoolwork, that person might appreciate an intervention aiming to redirect their focus away at least partly from idiosyncratic interests and towards school, even though the large majority of survey respondents were opposed to suppressing intense interests. However, working on these types of skills need not solely mean changing the autistic person’s behavior. It is also necessary to interrogate how the environment is leading to these issues and incorporate appropriate accommodations as necessary (e.g., wearing headphones if there are loud noises; utilizing principles of Universal Design for Learning to ensure an accessible and motivating curriculum; Armstrong, 2012). Ultimately, two of the most important things regarding goal identification is ensuring that they serve a purpose other than “normalization” and that they do not interfere with the autistic *way of being* (Schuck et al., 2021; Sinclair, 1993).

The third group of goals were *social* in nature. Most of these goals were endorsed by most participants (except for *improving school readiness* which was endorsed by 49.1%). However, the mean ratings were lower (ranging from 3.3 to 4.9) than those of the *uncontroversial* goals (which ranged from 4.8 to 5.4), indicating that these goals have the potential to be an important part of intervention programs, but may not be as relevant as those related to safety and quality of life. It might be an indication that these goals are more nuanced and that more information on specific skill areas is needed. For example, improving conversational skills has the potential to include a multitude of specific targets including reciprocal conversations, small talk, changing topics, and initiating questions. Communication skills are even broader and promoting them could involve anything from prioritizing verbal speech (rejected by most participants) to a flexible approach using any technique that works (strongly endorsed by most participants). Even if participants were not opposed to certain goals themselves, they might be concerned about *how* the goals are being implemented. Although research shows that building and maintaining friendships is important to autistic people (Mazurek, 2014; Sosnowy et al., 2019), curricula intended to explicitly teach social skills to autistic people sometimes fail to accurately prepare clients for the complex, dynamic, highly implicit nature of social interactions (Bottema-Beutel et al., 2018). Moreover, interventions focused on social interaction for autistic individuals typically focus on teaching them how to interact with neurotypical people (Bottema-Beutel et al., 2018; Sutton et al., 2019), ignoring the responsibility that neurotypical people have in creating positive social interactions with autistic people (known as the double empathy problem; Milton, 2012). This could lead to autistic individuals feeling that they constantly need to mask their autistic traits to fit in with neurotypical peers (Ai et al., 2022; Cook et al., 2021; Pearson & Rose, 2021) and further highlights that ABA interventions often focus on “normalization”, a major concern for autistic self-advocates. Thus, targeting social goals may be socially valid, but only if done so within the context of successful *autistic* social interaction (see the concept of *neurobilingualism;* Cerda, 2021a).

### Social Validity of Intervention Practices and Procedures

Most participants felt that clinicians should try taking the perspective of their autistic clients and focus on their thoughts and feelings as opposed to only observable behavior. Though this approach is becoming more popular (e.g., Cerda, 2021b), behavioral interventions are rooted in observable outcomes and behaviors, and they explicitly avoid the interpretation of “private events”, including thoughts and feelings (Heron et al., 2007). This is likely a contributing factor to the lack of social validity measures in many autism intervention studies (Callahan et al., 2017; D’Agostino et al., 2019; Snodgrass et al., 2018). The perspectives of the autistic adults offered in this study suggest that interventions consider shifting away from strict behaviorism and incorporate a stronger focus on clients’ internal experiences. The integration of more humanistic therapeutic practices (e.g., normalization of feelings, validation) could address the hyperfocus on behaviors that is evident in behavioral interventions. This suggestion could at least in part address the concern that ABA interventions prioritize compliance over quality of life (Sandoval-Norton & Shkedy, 2019). Being empathetic to the child’s internal feelings and normalizing and validating these experiences is incompatible with intervention that pushes for compliance above all else.

There was some indication that participants value the intervention components of more naturalistic intervention models. Specifically, many participants endorsed using the child’s interests to teach new skills, using the natural setting (e.g., the child’s home) as the intervention setting, and using natural reinforcement. These are core components of all NDBI models and represent the biggest theoretical differences between those and more traditional models, such as DTT. Most participants also endorsed interventionists engaging with autistic children by imitating their behaviors, which is another component of some NDBIs (e.g., ProjectIMPACT, Ingersoll & Dvortcsak, 2010; JASPER, Kasari et al., 2008; ESDM, Rogers & Dawson, 2010). This suggests that autistic adults may find some of the theoretical practices of NDBIs socially valid. Though limited, there was also some data to suggest that these natural components are preferred when directly compared to components of more structured models. Specifically, participants preferred natural reinforcement over external reinforcement and interventions that occur in the natural setting over both clinic and school settings. However, parent-delivery of interventions was not preferred over clinician-delivery, though it is common practice in the use of NDBIs to teach parents to implement these interventions. As such, it is not overwhelmingly clear that *all* aspects of NDBIs are preferred from the perspective of autistic adults. Moreover, as all components of naturalistic and more structured models were not compared, and as this study does not systematically compare ABA-based to non-ABA intervention procedures, more research is warranted in this area to determine the extent to which autistic adults might prefer certain intervention models over others.

Another clear preference was around the topic of communication modalities. Participants indicated that they preferred the promotion of any communication method over focus on verbal communication. In fact, 99% of participants endorsed the statement *interventionists should promote communication using any technique that works (for example, sign language, pointing to symbols, speech generating devices).* This overwhelming consensus demonstrates that allowing autistic individuals to select their preferred communication modality is highly valued within the autistic community. The push for vocal speech is just one example of how neurotypical standards of what is “normal” take precedence over autistic needs. Notably, while a substantial amount of NDBI research focuses on improving ‘social communication’ and ‘language’ skills (Crank et al., 2021), few NDBI studies explicitly focus on using alternative communication modalities (Gevarter & Zamora, 2018). Therefore, even though components of NDBIs may be socially valid, the prioritization of vocal speech within the intervention context might not be.

While this survey sought to gain more insight into socially valid intervention practices and procedures, this information is not necessarily representative of the feelings of all autistic people. Therefore, it does not replace the need for additional research evaluating the social validity from the perspective of additional autistic populations, nor does it replace the need for individualized autistic input. Participants overwhelmingly indicated that autistic adults should be consulted when developing intervention goals and autistic children should play a role in developing their own goals when possible (96% and 95% respectively). Children choosing their own goals was also preferred over both parent-chosen (62%) and professional-chosen (65%) goals, though it should be emphasized that all of these items were endorsed by a majority of respondents. This suggests that participants may have valued a collaborative approach, with parents, professionals, autistic children, and autistic consultants all providing important insights in intervention goal development. From this, we believe autistic consultancy should be common practice in intervention settings if autistic consultants are fairly compensated for their time and insights, perhaps analogously to compensation for autistic community partners in participatory studies (see, e.g., den Houting et al., 2021; Nicolaidis et al., 2019). Given that this study is one of few evaluating social validity of intervention goals and procedures from the perspective of autistic persons, individualized assessments of social validity are arguably even more important, as limited information is currently available.

### Limitations and Future Directions

Though this study provides a starting point for understanding the views of autistic adults on common intervention goals, procedures, and practices, there are several limitations to address. First, the AIAS is an ad-hoc measure created specifically for this study, as other instruments to evaluate the social validity of the goals and procedures of behavioral interventions in this way were not available. However, this measure has not yet been subjected to rigorous validation work. For example, we did not conduct cognitive interviews with potential participants to gauge their thought process while answering AIAS questions. Thus, it is possible changes in item wording may lead to different findings (e.g., using the word “support” may have led to less strong reactions than “intervention,” as “intervention” invokes a medical model approach). Thus, our results should not be regarded as the final organization of goals as recommended by autistic adults. Instead, they should be seen as a preliminary investigation into the degree to which certain goals might be considered more socially valid and others not. Thus, validating this measure should be a priority for future iterations of this work. Additionally, the AIAS assessed only two out of the three domains of social validity (goals and procedures, but not outcomes). It will be worthwhile for future investigations using the AIAS or similar measures to include questions that tap into all aspects of social validity. However, the information gathered in this preliminary investigation remains an important contribution. At this time, there is little available on this topic in the academic literature, but autistic opinions on behavioral interventions are available in non-academic forums (e.g., blogs, social media). The results of this study are in line with these critiques, as well as other emerging research on goal acceptability (Waddington et al., 2023), suggesting at least some validity in our results.

Second, those most likely to complete such a survey are likely interested in this topic, feel they have something to share, and can communicate via a survey, which indicates some sampling bias. Additionally, due to the online nature, the sample is limited to those with the means and ability to access an online survey and those who can read and fully understand the intent of the questions. Similarly, study participants overwhelmingly identified as White and female, which is not fully representative of the autistic population, particularly the subset who receive behavioral intervention. It is possible that individuals from other demographic groups would have differing opinions on intervention goals, procedures, and practices. Additionally, a relatively small proportion of the sample (under 30%) reported experiencing behavioral intervention in childhood, which is probably related to the fact that the mean age of diagnosis amongst our participants was approximately 24 years old. Thus, our participants likely received fewer services/interventions than most autistic individuals, particularly during childhood, and views of autistic individuals who experienced ABA-based interventions or other types of support could have been underrepresented. This also relates to the sample bias and therefore these results should be interpreted cautiously as some participants’ responses were less informed by direct experience than others. However, it is notable that receipt of prior behavioral intervention was not significantly associated with goal endorsement in any of the three clusters. Moreover, it should be noted that due to the nature of the survey format, it is likely that individuals surveyed had average or above average intellectual functioning, which inadvertently excludes a large portion of the autistic population that currently receives these services. Additional work needs to be done to evaluate the perspectives of this important group of intervention users. Further exploration of this topic needs to ensure a diverse sample through explicit recruitment of underserved populations and individuals with lived experience of these interventions.

Another limitation of the study is the broadness of some of the statements about intervention goals. Communication goals and social skills goals are broad goal areas that encompass a plethora of potential skill targets that vary with developmental level. As these are often the main focus of interventions, additional research is needed to obtain more feedback on specific communication and social skills targets. Plus, participants were not asked for their opinions on targeting play skills, and this should be included in future iterations of the AIAS given that autistic individuals in Waddington and colleagues’ (2023) study ranked play goals as low priority. AIAS survey items were also provided with no context as to the rationale or purpose behind these goals and practices. Because all supports must be individualized for each recipient, it is possible that providing more details about when and for whom a goal might apply would affect participants’ answers (for example, a school-readiness goal may be seen as more acceptable for an older child, but not a toddler). Additionally, these data do not capture participants’ rationale for their endorsements of these items. Further survey measures and in-depth interviews would be beneficial in continuing to gather more information on this topic.

Moreover, it would be helpful to identify the perspectives of autistic children who are currently participating in behavioral intervention programs that utilize these goals and procedures. Innovative methods exist for capturing perspectives of autistic people who face barriers to conventional surveys and interview techniques due to cognitive or communication difficulties (e.g., Courchesne et al., 2021; Do et al., 2021; Kirby et al., 2015). Combining the opinions of autistic adults and children will strengthen the findings of which practices and goals are socially valid. With more focus on research that prioritizes these perspectives, clinicians, teachers, and parents will be able to make more informed intervention decisions and begin to reform behavioral interventions, such that they value the perspectives of autistic people above all else.

There are many potential issues surrounding community-based implementation of these interventions that cannot be addressed in this study. First, there might be many ways of pursuing particular intervention goals with varying levels of acceptability. Additionally, there can often be gaps between research and community implementation of a given intervention. Indeed, many behavior interventionists appear to lack information regarding NDBIs (Hampton & Sandbank, 2022; also see Stahmer et al., 2005, 2012). Unfortunately, it is unclear how aware most community ABA practitioners are of autistic community concerns regarding ABA, but based on anecdotal observations, we believe there are likely substantial knowledge gaps in that area as well.

Until additional research investigates outstanding questions regarding social validity of behavioral interventions, it is possible that, if an intervention uses components that appear to be socially valid based on feedback from autistic adults in the present study, the implementation of additional safeguards—such as hiring autistic adults to provide consultation and supervision—could be sufficient to ensure a high level of social validity (Schuck et al., 2021). However, such consultation is still a mostly theoretical consideration and has not been implemented widely or systematically enough to determine what best practice is for incorporation of autistic perspectives into ABA practice. Future research should engage interested parties such as autistic adults, autistic and non-autistic parents, and intervention agencies to explore models of how autistic consultancy could be implemented on a larger scale.

## Conclusion

Though behavioral interventions have been used with autistic children for decades, assessing the social validity of such programs has not been prioritized (Callahan et al., 2017; D’Agostino et al., 2019; Ledford et al., 2016), and many autistic people described what they believe are flaws in both the goals of intervention and their common practices. This study helps identify which common intervention goals, practices, and procedures are socially valid from the perspectives of autistic adults, which are perceived as problematic, and which require more investigation. Our findings show that participants did overwhelmingly endorse many common intervention goals, specifically those that focused on overall quality of life and safety, as well as common intervention strategies, namely those that emphasized individualization, use of natural intervention strategies, and taking individuals’ feelings and preferences into account. Results also indicate that participants clearly do not support certain goals and practices, particularly those that prioritize normalization (e.g., reducing vocal/physical stimming and fixations, promoting communication by focusing primarily on verbal speech). These findings can be used as a starting point to help guide clinicians and educators toward designing intervention goals and using intervention practices and procedures that are seen as socially valid by autistic individuals, which will hopefully lead to more neurodiversity-affirming interventions that address the concerns of the autistic community.

## Footnotes

1 The term “rewards” was used instead of “reinforcement” in the survey items to increase understanding, as the behavioral definition of reinforcement is not widely known outside of the ABA field. While by definition, these are not synonymous with one another, colloquially they are often used interchangeably. Therefore, the use of “reinforcement” is used in discussions throughout the paper to stay in line with the behavioral literature, but “rewards” is used when specifically referring to wording of survey items to remain in line with what participants responded to.
